# Renovation as innovation: Repurposing human antibacterial peptide LL-37 for cancer therapy

**DOI:** 10.3389/fphar.2022.944147

**Published:** 2022-08-23

**Authors:** Fatai Lu, Yingkang Zhu, Guodong Zhang, Zunpeng Liu

**Affiliations:** Department of Orthopedics, The Fourth Affiliated Hospital of China Medical University, Shenyang, China

**Keywords:** antimicrobial peptides, cathelicidin (LL37), hCAP18, LL37, cancer, anticancer

## Abstract

In many organisms, antimicrobial peptides (AMPs) display wide activities in innate host defense against microbial pathogens. Mammalian AMPs include the cathelicidin and defensin families. LL37 is the only one member of the cathelicidin family of host defense peptides expressed in humans. Since its discovery, it has become clear that they have pleiotropic effects. In addition to its antibacterial properties, many studies have shown that LL37 is also involved in a wide variety of biological activities, including tissue repair, inflammatory responses, hemotaxis, and chemokine induction. Moreover, recent studies suggest that LL37 exhibits the intricate and contradictory effects in promoting or inhibiting tumor growth. Indeed, an increasing amount of evidence suggests that human LL37 including its fragments and analogs shows anticancer effects on many kinds of cancer cell lines, although LL37 is also involved in cancer progression. Focusing on recent information, in this review, we explore and summarize how LL37 contributes to anticancer effect as well as discuss the strategies to enhance delivery of this peptide and selectivity for cancer cells.

## Introduction

As the key components of the innate host immune system, antimicrobial peptides (AMPs) have been discovered in almost all life forms, ranging from bacteria to higher mammals, and act as primary defense against a broad spectrum of pathogens (Jafari et al., 2022). Mammalian AMPs include the cathelicidin and defensin families. Cathelicidins possess a highly conserved cathelin-like prosequence and variable carboxyl-terminal sequences that are consistent with the mature AMPs (Johansson et al., 1998). The only member of cathelicidin identified in humans is hCAP18, which is a positively charged antibacterial protein, with a molecular weight of 18 kDa. LL-37 is released as an active domain of hCAP18 through extracellular cleavage mediated by proteinase-3 enzyme (Kuroda et al., 2015a).

A number of studies have reported that LL-37 exerts a diverse range of pleiotropic attributes including antimicrobial activities, immunity, angiogenesis, wound repair, and bone tissue engineering (Tjabringa et al., 2003; Elssner et al., 2004; Bucki et al., 2010; Pfosser et al., 2010; Ramos et al., 2011; Liu et al., 2018; Mitchell et al., 2022). However, different from its traditional roles, emerging evidence from cancer biology studies suggests that LL-37 might promote or inhibit tumor progression (Ren et al., 2012; Piktel et al., 2016; Chen et al., 2018; Jiang et al., 2020; Chen et al., 2021; Vitale et al., 2021; Kiatsurayanon et al., 2022; Zhang et al., 2022). LL-37 plays an important and complex role in the regulation of different human cancers. These data are beginning to reveal the complex and contradictory functions of LL-37.

In this review, we first introduce the characteristic features of LL-37, focusing on its anticancer effects on various human cancers and the underlying mechanisms involved. Based on the recent studies, we also discuss the therapeutic implications of LL-37 as a potential anticancer drug. We believe that this important peptide will eventually be developed into a new anticancer drug suitable for clinical use in the future.

## Characteristics and structure of LL-37

### Characteristics of LL-37

**FIGURE 1:**
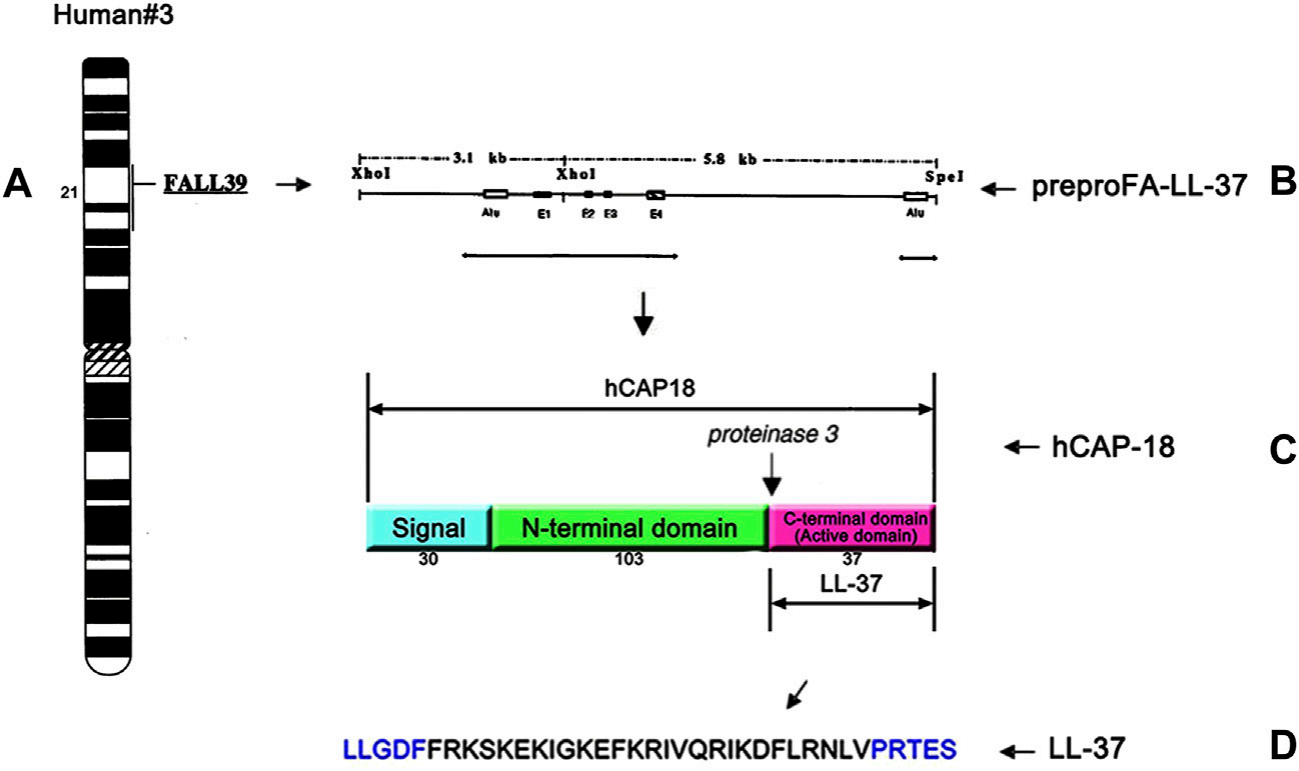
Single cathelicidin gene called *CAMP* located on human chromosome 3p21.3 encodes hCAP18 **(A)**, a schematic drawing of cDNA for the complete prepro-LL-37 **(B)**, structure and cleavage sites of hCAP18 **(C)**, and the amino acid sequence of the antibacterial peptide LL-37 **(D)**. The human cathelicidin hCAP18 consists of a signal peptide (30 amino acids), N-terminal domain (103 amino acids), and C-terminal domain (37 amino acids). The C-terminal domain shows various activities as an active domain and is called LL-37.

Human AMPs include the cathelicidin and defensin families. Different from other animals, there is only one cathelicidin gene in humans (Frohm et al., 1997; Zanetti, 2005). As shown in Figure 1, the single *cathelicidin* gene called *CAMP* located on the human chromosome 3p21.3 encodes the human cationic antimicrobial peptide-18 (hCAP18) which is composed of 170 amino acids (Zanetti, 2004; Dürr et al., 2006). Like most antimicrobial peptides, hCAP18 is also produced as inactive preproproteins. It is a major component of the azurophilic granules of the neutrophils (Cowland et al., 1995; Sørensen et al., 2001) and is primarily produced by bone marrow, keratinocytes of inflamed sites, and cells of the mucosal epithelium (Agerberth et al., 1995; Chen and Fang, 2004; Tjabringa et al., 2005; Wolk et al., 2006). Once cell injury or infection occurs, it can provide a trigger to activate the cell degranulation by stimulating toll-like receptors (TLRs) and/or altering the cytokine (Vandamme et al., 2012). Thereafter, the inactive hCAP18 precursor protein is released from the intracellular environment and then processed by the proteolytic cleavage into the active LL-37 peptide (Zaiou et al., 2003; Fahy and Wewers, 2005; Pazgier et al., 2013).

LL-37 (4.5 kDa) is an active 37-amino acid peptide. The precursor protein pre-hCAP18 (18 kDa) is converted into propeptide hCAP18 (16 kDa) *via* processing of the signaling peptide, and then the active LL-37 peptide is produced from the C-terminus of hCAP18 *via* specific serine proteases, for instance, proteinase 3 (PR3) (Vandamme et al., 2012; Gudmundsson et al., 1996). Its primary sequence is LLGDFFRKSKEKIGKEFKRIVQRIKDFLRNLVPRTES (Gudmundsson et al., 1996). LL-37 is commonly found in mucosal secretion, sweat, semen, urine, breast milk, and plasma (Malm et al., 2000; Murakami et al., 2002; Armogida et al., 2004; Rieg et al., 2005; Berkestedt et al., 2010; Fábián et al., 2012; Babikir et al., 2018).

### Structure of LL-37

**FIGURE 2:**
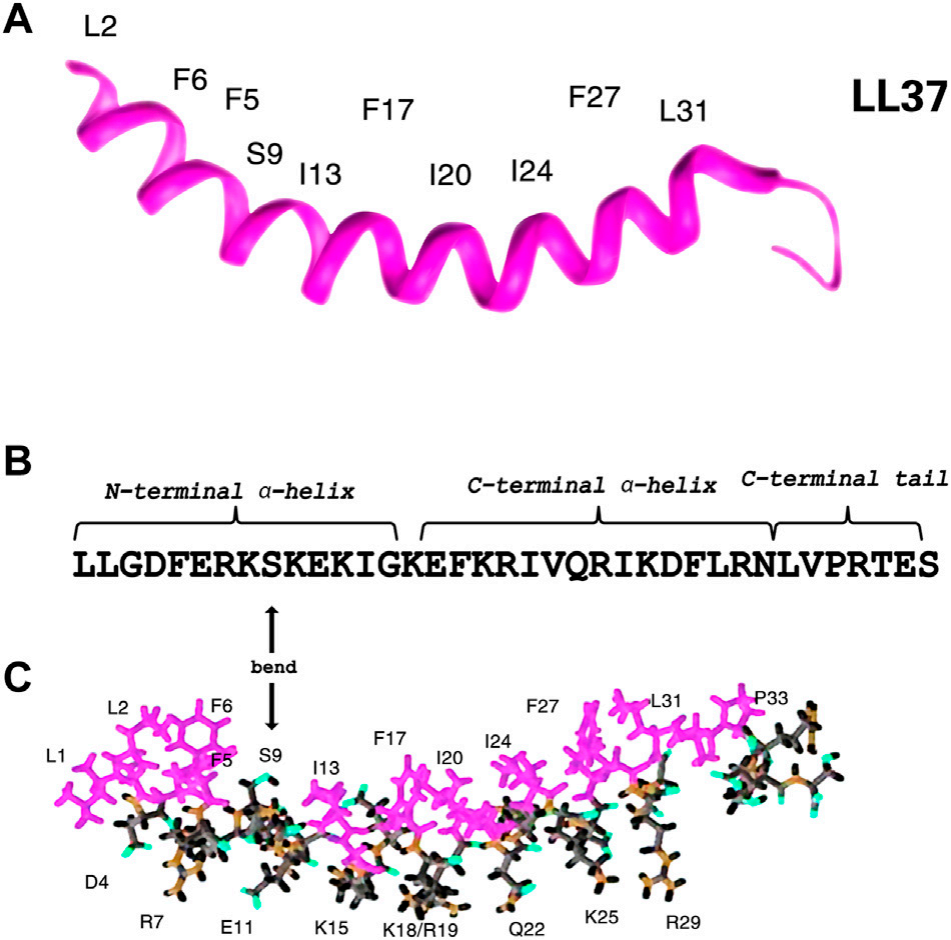
Three-dimensional structure of human cathelicidin reveals a helix followed by a C-terminal tail. Note the four phenylalanine side chains lying on the concave surface of the peptide (F6, F5, F17, and F27) **(A)**. Sequence of LL-37. S9 is marked **(B)**. Stick view of the structure of LL-37 with hydrophobic and hydrophilic residues selectively labeled. In both views, hydrophobic amino acids are in purplish red **(C)**. Therefore, it is evident that there is a discontinuation of the hydrophobic surface at S9 rather than the helices (Wang, 2008).

According to previous circular dichroism (CD), Fourier transform infrared (FT-IR) (Tossi et al., 1994; Oren et al., 1999) spectroscopy, and NMR spectroscopy studies (Porcelli et al., 2008; Wang, 2008; Wang et al., 2014), LL-37 possesses a linear cationic *α*-helical structure which might aid to exert its function. As shown in Figures 2A,B, the *α*-helical structure spanning residues 2 to 31 with unstructured C-terminal residues 32 to 37 consists of three parts, namely, an N-terminal *α*-helix with a pair of leucine residues (LL), a C-terminal *α*-helix, and a disordered C-terminal tail (residues 32–37) (Porcelli et al., 2008; Wang, 2008; Wang et al., 2019). The C-terminal tail is mobile, while the helical region is rigid. LL-37 is bent with a series of hydrophobic side chains, whereas its hydrophobic surface bordered by the positively charged residues is composed of four distinct aromatic phenylalanine side chains that all point in the same direction (Wang, 2008; Vandamme et al., 2012). The amphiphilic peptide with a positive charge and net charge of +6 can facilitate an interaction with the negatively charged molecules or structures, such as bacterial cell walls (Wang, 2008). Similarly, LL-37 also targets and binds to the cancer cells as the anionic phosphoryl serine is exposed on their surface (Wu et al., 2010a). Interestingly, not only antimicrobial but also the anticancer effect is primarily exerted by the C-terminal helix (Li et al., 2006; Falcao et al., 2015). Moreover, the N-terminal helix has been related to hemolytic activity, proteolytic resistance, and chemotaxis, whereas the disordered C-terminal tail is essential for tetramerization (Wang, 2014). The two helices are separated by a bend or break. Furthermore, it has been found that the discontinuation is found on the hydrophobic surface at S9, rather than the helix (Figures 2B,C) (Wang, 2008; Zhang et al., 2021).

## How LL-37 can eradicate/affect cancer?

The cytotoxic effects of numerous AMPs on different tumor cell lines have been reported previously (Cheng et al., 2020; Lee et al., 2021; Athira et al., 2022; Jafari et al., 2022; Patil and Kunda, 2022). These AMPs contain several cationic and hydrophobic amino acids and were found to be involved in various anticancer activities. They were thus termed as anticancer peptides (ACPs) (Hoskin and Ramamoorthy, 2008). ACPs can bind and kill the cancer cells through direct or indirect mechanisms (Dennison et al., 2006; Huang et al., 2015).

ACPs exert their biological functions in a diverse manner. These ACPs generally contain positively charged amino acids like lysine and arginine and possess a net positive charge ranging from +1 to +9 at neutral pH (Habes et al., 2019; Chiangjong et al., 2020). Moreover, as AMPs bind with bacterial membranes, ACPs can bind directly with the cancer cell walls due to their cationic and amphipathic nature (Ma et al., 2019). It has been established that different from normal eukaryotic cell membranes which are made of uncharged neutral phospholipids, sphingomyelins, and cholesterol and are neutral in charge (Zachowski, 1993; Doktorova et al., 2020), the surface of the cancer cells is net negatively charged because of increased proportions of anionic phosphatidylserine, heparan, and chondroitin sulfate proteoglycans, O-glycosylated mucins, and sialylated glycoproteins (Warren, 1974; Warren et al., 1979; Utsugi et al., 1991; Zwaal et al., 2005; Calianese and Birge, 2020; Brockhausen and Melamed, 2021; Hassan et al., 2021; Hugonnet et al., 2021). ACPs can selectively recognize cancer cells by electrostatic interactions with the negatively charged phospholipids on the surface. Some ACPs tend to kill cancer cells by causing membrane perturbation; however, some ACPs can penetrate the target cell and disrupt the mitochondrial membrane, thereby resulting in apoptosis (Deslouches and Di, 2017). ACPs bind to the membranes in different models, including carpet model, surface binding non-inserted, and perpendicular to the surface (Quemé-Peña et al., 2021). ACPs can enter the cells through two distinct mechanisms: direct or indirect. The former causes irreparable membrane damage, followed by the cell lysis, which is non-energy dependent, and the latter can modulate the integrity of the cancer cell membrane by altering some intracellular pathways, thereby resulting in cell death by apoptosis, which is energy dependent (Kumar et al., 2018; Hilchie et al., 2019; Jafari et al., 2022).

One of the best-studied ACPs is LL-37. However, contradictory results have been shown for LL-37 linked to cancers in different models. The existing data indicate that LL-37 can exert a tumorigenic effect in some cancers, including lung cancer, breast cancer, ovarian cancer, melanoma, prostate cancer, liver cancer, and skin squamous cell carcinoma (Coffelt et al., 2009; Cha et al., 2016; Muñoz et al., 2016; Wang et al., 2017; Habes et al., 2019; Jiang et al., 2020; Ding et al., 2021; Zhang et al., 2022). Mechanistically, LL-37 activated Wnt/β-catenin signaling by inducing the phosphorylation of protein kinase B and subsequent phosphorylation of glycogen synthase kinase 3β mediated by the toll-like receptor-4 expressed in lung tumor cells (Ji et al., 2019). Furthermore, LL-37 cooperated with IL-33 to increase the phosphorylation of p38 MAPK and NF-κB p65 pathways and augmented IL-6 and IL-1β secretion, which resulted in the proliferation of lung cancer cells. Sulfated glycoaminoglycans and proteoglycan syndecan-4 increase the binding of LL-37 to the cell surface, which promotes the migration of breast cancer cells. In addition, *via* activating TRPV2 and PI3/Akt signaling, and then inducing recruitment of TRPV2 from intracellular vesicles to the plasma membrane of pseudopodia, LL-37 promotes proliferation and growth of breast cancer cells (Farabaugh et al., 2016). On the contrary, it has also been shown that LL-37 can exert anticancer effects on other cancers, including colon cancer, glioblastoma, hematologic malignancy, gastric cancer, and oral squamous cell carcinoma (Aarbiou et al., 2006; Wu et al., 2010b; Bruns et al., 2015; Prevete et al., 2015; Chen et al., 2020; Porter et al., 2021; Chernov et al., 2022). There is no smoking gun to explain the reported opposite effects on different cancer types. Whether and how LL-37 can affect cancer and metastasis deserves further studies. In the next section, our principal discussion focusses on the potential anticancer mechanisms of LL-37.

### The membranolytic mechanisms

**FIGURE 3:**
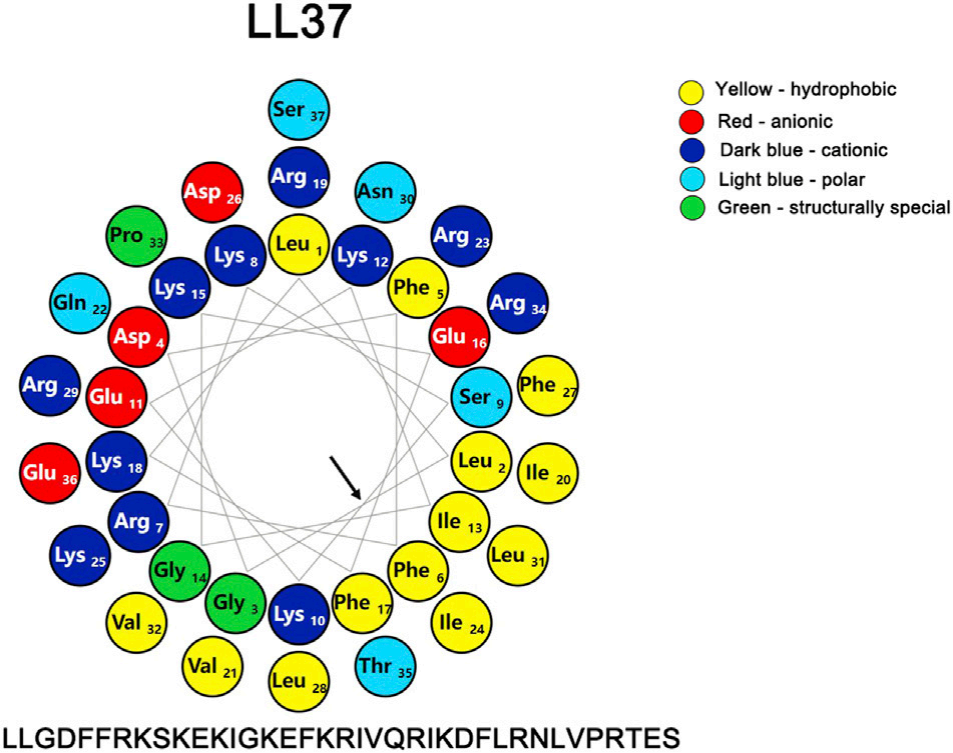
Helical wheel representation of LL-37, illustrating the amphipathic and cationic nature of LL-37. The residues are color coded: potentially negatively charged as red, potentially positively charged as dark blue, the hydrophobic residue is in yellow, the polar residues are coded as light blue, and the structurally special residues are coded as green. The helix diagram of the polypeptide was drawn with a Protein ORIGAMI (Reißer et al., 2018) software package. The arrow indicates the hydrophobic surface of the peptide.

LL-37 could directly bind and perturb efficiently zwitterionic PC (phosphatidylcholine) and negatively charged PC/PS (phosphatidylcholine/phosphatidylserine) phospholipid membranes (Juba et al., 2015). The initial interaction with the membrane is primarily brought about by various electrostatic forces, and the correlation between the cationic charge and biological activity is strengthened with the increasing charge until the optimum charge for activity has been reached (Fillion et al., 2015; Juba et al., 2015). The presence of the negatively charged lipids such as anionic phosphatidylserine (PS) in membranes of the cancer cells can also mediate an electrostatic interaction with the cationic peptides (Alvares et al., 2017; Vasquez-Montes et al., 2019). In addition to its high net positive charge (+6) (Figure 3) that can markedly reduce the repulsive forces *via* neutralization by the negative charges, the high affinity of LL-37 for the negatively charged membranes in light of its hydrophobic interactions between the peptide and the membranes has been reported (Oren et al., 1999; Shai, 2002).

A number of studies (Pouny and Shai, 1992; Oren et al., 1999; Ding et al., 2013; Wang, 2015; Lee et al., 2016; Zhao et al., 2018) have shown that, different from other ACPs, the model of action of LL-37 with negatively charged membranes such as the membranes of the cancer cells is a detergent-like effect exhibited through a “carpet-like” mechanism rather than a channel-forming model. In contrast to the channel formation mechanism, when bound to either zwitterionic PC or negatively charged PC/PS, LL-37 can effectively dissociate into monomers, and the hydrophobic N-terminus of LL-37 is buried only slightly in the membrane.

**FIGURE 4:**
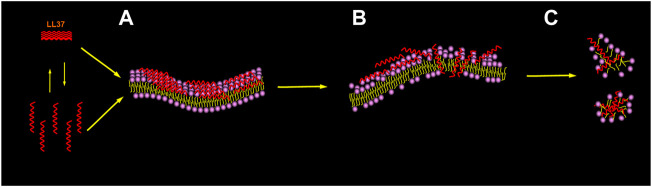
Membrane-associated mechanism for the peptide. Picture illustrating the carpet model recommended for membrane permeation. The initial binding to the membrane interface is mediated by the electrostatic interaction. The peptide reaches the membrane in the form of a monomer or oligomer and then binds to the membrane surface **(A)**. When the threshold concentration of peptide monomer is reached, the membrane is penetrated and forms instantaneous pores **(B)**, which also leads to membrane disintegration **(C)**.

Specifically (Oren et al., 1999; Shai, 2002; Lee et al., 2016; Quemé-Peña et al., 2021), as shown in Figure 4, LL-37 reaches and remains on the negatively charged membranes such as the membranes of cancer cells as oligomers of different sizes; thereafter, a change in the membrane energetics and fluidity causes several local perturbations followed by dissociation into the monomers. Afterward, it is bound to the surface of the membrane, with the hydrophobic surface facing the membrane and the hydrophilic surface facing the solvent. When the threshold concentration is reached, the peptide monomers can easily diffuse into the membrane, cover, and disintegrate it in a detergent-like manner through a “carpet-like” mechanism. The overall outcome can lead to cancer cell death, such as that reported in acute myeloid leukemia cells (Xhindoli et al., 2014), bronchial epithelial cancer cells (Tzitzilis et al., 2020), and human osteosarcoma cells (Bankell et al., 2021a).

### The non-membranolytic mechanisms

**FIGURE 5:**
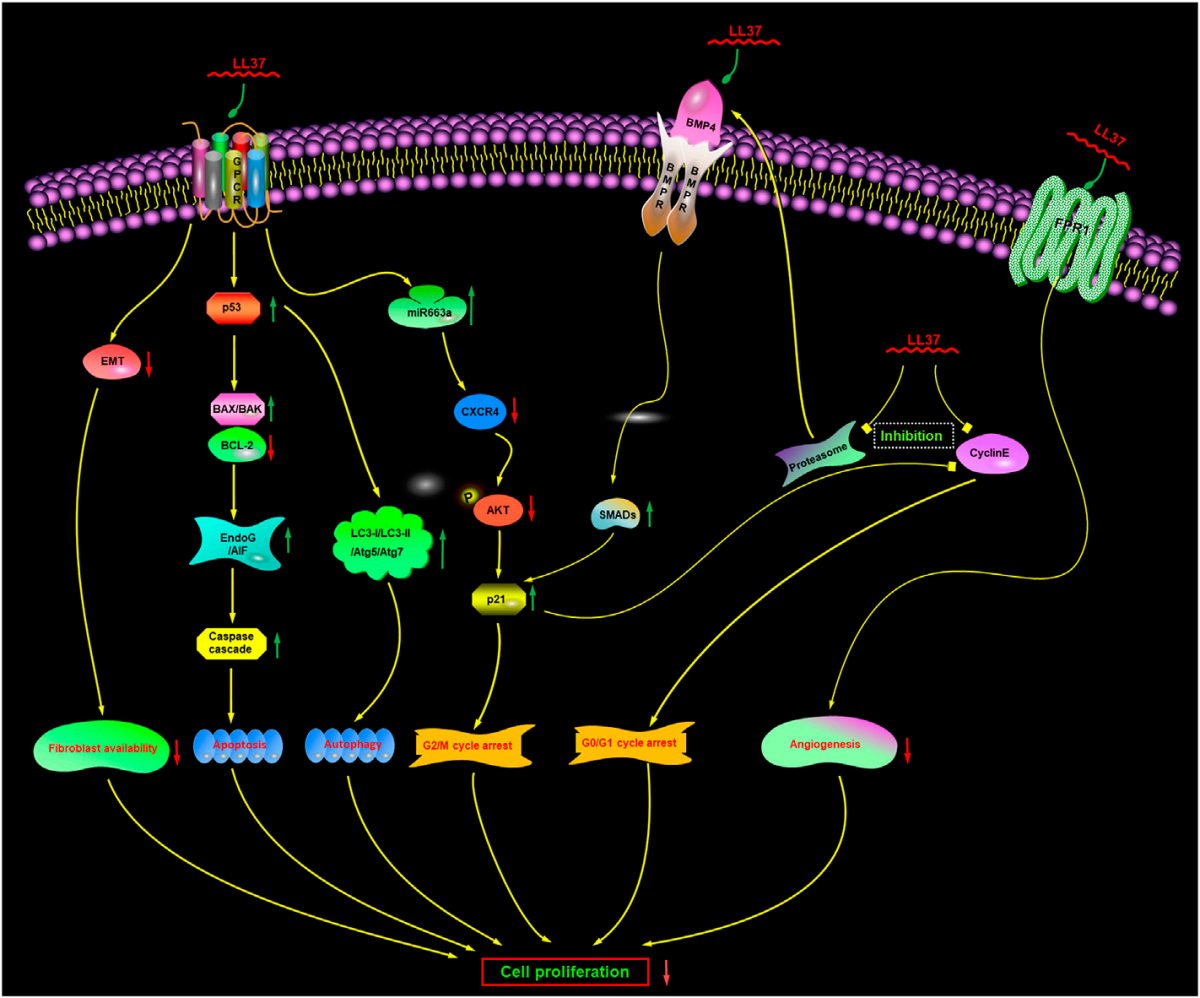
Proposed non-membranolytic anticancer mechanism of human cathelicidin LL-37. Inhibition of proteasome activity induces the upregulation of BMP4, which subsequently activates BMP signaling. GPCR, G protein-coupled receptor; CXCR4, CXC chemokine receptor type 4; EndoG, endonuclease G; AIF, apoptosis-inducing factor; FPR1, formyl peptide receptor 1. BMP4, bone morphogenetic protein 4; BMPR, bone morphogenetic protein receptor.

It was originally thought that membranolytic mechanisms were the only mechanism of action, but there is increasing evidence now to suggest that there may be also additional or complementary non-membranolytic mechanisms (Figure 5), such as a receptor-mediated mechanism.

#### G protein-coupled receptors

G protein-coupled receptors (GPCRs) are membrane-embedded receptors that can regulate several important biological functions. In some cancer cells (Mader et al., 2009; Ren et al., 2012; Piktel et al., 2016), LL-37 induces characteristic apoptotic cell death in a caspase-independent manner, such as phosphatidylserine externalization and DNA fragmentation, without activation of caspases. One requirement for caspase-independent apoptosis of cancer cells is the altered activity of Bcl-2 and p53. LL-37 has been reported to reduce the level of antiapoptotic Bcl-2 and increase the level of pro-apoptotic Bax/Bak (Mader et al., 2009; Ren et al., 2012; Ren et al., 2013; Chen et al., 2020; Yang et al., 2021). LL-37 can also increase the expression of p53 and p53-upregulated modulator of apoptosis (PUMA) (Ren et al., 2012; Piktel et al., 2016; Chen et al., 2020). PUMA, a direct transcriptional target of p53, is a highly efficient pro-apoptotic protein and acts as a modulator of apoptosis in several cancer cell lines (Han et al., 2001; Yu et al., 2001; Jeffers et al., 2003; Yu et al., 2003; Yu and Zhang, 2003; Roufayel et al., 2022). Another requirement for the caspase-independent apoptosis of cancer cells is the upregulated expression and translocation of apoptosis-inducing factor (AIF) and endonuclease G (EndoG). After treatment with LL-37, the nuclear levels of both AIF and EndoG are prominently increased and translocated from the mitochondria into the nucleus, resulting in cancer cell apoptosis that is caspase-independent but calpain- and AIF-dependent apoptosis and mediated *via* BAX activation (Mader et al., 2009; Ren et al., 2012; Açil et al., 2018; Bankell et al., 2021b).

Nevertheless, interestingly, recent studies have suggested that except in a caspase-independent manner, the cell apoptosis induced by LL-37 can also occur through a caspase-dependent manner (Açil et al., 2018; Chen et al., 2020) *via* the p53-Bcl-2/BAX signaling pathway.

So, a mechanism was inferred that LL-37 can potentially exert its apoptogenic action in a caspase-independent or caspase-dependent manner *via* activating a GPCR-p53-Bax/Bak/Bcl-2 signaling cascade to trigger AIF/EndoG-mediated apoptosis.

#### Regulation of the proteasome activation *via* bone morphogenetic protein signaling

The bone morphogenetic protein (BMP) signal is an important tumor suppressive pathway involved in the process of tumorigenesis. It is initiated *via* the binding of BMP ligands to BMP receptors, which can then recruit and phosphorylate the downstream Smad1/5/8. Thereafter, the heterodimers are formed by phosphorylated Smads with Smad4, which can translocate into the nucleus as transcription factors to induce the transcription of various genes mediating the biological effects of BMPs (Varga and Wrana, 2005). The proteasome is a multimeric protein complex with proteolytic activity, which can effectively upregulate the level of BMP ligands and stimulate the phosphorylation of Smad1/5/8 (Wu et al., 2008a; Wu et al., 2008b; Zhang et al., 2014).

The anticancer effect of LL-37 has been reported to involve regulation of the proteasome activation *via* modulation of BMP signaling (Rajkumar et al., 2005; Wu et al., 2010b; Wu et al., 2010c). The chymotrypsin-like and caspase-like activities of 20S proteasome have been reported to be significantly inhibited by LL-37. The expression of BMP4 and the phosphorylation of Smad1/5 are upregulated, and then the expression of p21^Waf1^ is subsequently induced at both the protein and mRNA levels (Rajkumar et al., 2005; Wu et al., 2010b). Furthermore, RNA interference which can target BMP receptor II was found to partially block the activation of the BMP signal and the inhibition of cell proliferation induced by LL-37. Moreover, LL-37 can also downregulate the expression level of cyclin E2 (Wu et al., 2010b). Both p21WAF1 and cyclin E2 can regulate the cell cycle progression by affecting the late G1 phase (Bartek and Lukas, 2001). As shown in Figure 5, the alteration of p21 and cyclin E2 expression levels can trigger G0/G1 phase cell cycle arrest and contribute to the antitumor effects of LL-37 (Wu et al., 2010a; Wu et al., 2010b). Furthermore, MG-132, the proteasome inhibitor, can produce similar effects to those of LL-37. It can induce the BMP/p21 cascade to inhibit cell proliferation in the gastric cancer cells. However, the inhibition of cancer cell proliferation could not be blocked by pertussis toxin. These findings clearly suggested that LL-37 could exert its anticancer effects through the activation of BMP signaling *via* a proteasome-dependent mechanism (Wu et al., 2010b).

### LL-37 can act as an antitumor immunostimulatory agent on the host immune system

Immune modulation and anticancer activity are the two different faces of the same coin. A recent study has conclusively demonstrated that LL-37 can significantly influence immune responses as an essential component of innate immunity (Yang et al., 2020). Aside from the anticancer activity of LL-37, the immunostimulatory or adjuvant effect has also been used. CpG-oligodeoxynucleotides (CpG-ODNs), a toll-like receptor TLR9 ligand, are employed to enhance the tumor suppressive activity of the host immune cells in immunotherapy (Wu et al., 2010a). It has been shown that LL-37 can markedly enhance the perception of CpG-ODN and then induce the proliferation and activation of the host immune cells, such as natural killer (NK) cells, plasmacytoid dendritic cells, and B lymphocytes. These cells can thereafter induct and maintain antitumor immune responses and mediate tumor destruction (Chuang et al., 2009; Büchau et al., 2010; Hurtado and Peh, 2010).

Furthermore, it has been shown that LL-37 can act and expand OVA-antigen-specific CD8^+^ T cells in draining the lymph nodes and the tumor microenvironment (Mader et al., 2011a; Singh et al., 2012), which could potentially delay tumor growth. LL-37 can also promote an anticancer immune response *via* inhibiting CD25^+^CD4^+^FOXP3^+^T regulatory cells (Mader et al., 2011b). Moreover, some studies have demonstrated that intra-tumoral injections of LL-37 stimulate the innate immune system by acting plasmacytoid dendritic cells, which can in turn mediate tumor destruction (Dolkar et al., 2018). In fact, LL-37 has been utilized in a phase 1 clinical trial for melanoma patients with cutaneous metastases *via* intra-tumoral injections. These findings suggested that LL-37 could be employed as an antitumor immunostimulatory agent and could provide a promising strategy for antitumor immunotherapy.

## Strategies to enhance LL-37 delivery and selectivity for cancer cells

Since both anticancer and cytotoxic activities of LL-37 are inhibited in human plasma, the delivery platform and modification strategies might be needed to ensure that LL-37 can reach the tumor microenvironment and promote tumor cell targeting, such as the use of nanoparticles and fusogenic liposomes and the design of peptides (Wang et al., 1998; Hilchie et al., 2019; Wang et al., 2019).

### Use of nano-sized drug delivery systems

Application of nano-sized drug delivery systems can serve as a potential strategy to improve the delivery of peptides into host cells (Radaic et al., 2020). Nanoparticles with different structures and materials have been examined previously to facilitate the optimal delivery of anticancer peptides (Marverti et al., 2020; Akkın et al., 2021; Zielińska et al., 2021). In addition to being stable and non-toxic, the nanoparticles must be targetable in order to facilitate directed delivery of drugs to the exact tissues or cells (Hilchie et al., 2019). For instance, it has been reported that LL-37 loaded onto zinc oxide nanoparticles (ZnO NP) significantly suppressed the growth of the human lung cancer model cell line (BEAS-2B) (DeLong et al., 2019). Moreover, LL-37-loaded thermosensitive hydrogel nanoparticles displayed improved antiangiogenesis and antitumor activity (Fan et al., 2015). Moreover, it has been shown that CaP nanoparticles also can protect LL-37 from proteolysis (Tsikourkitoudi et al., 2020). Moreover, as reported in the literature, the anticancer activity of LL-37 improved when loaded onto the magnetic nanoparticles (Niemirowicz et al., 2015; Niemirowicz et al., 2017; Wnorowska et al., 2020).

Liposomes are lipid-based nanoparticles. Hydrophobic or hydrophilic drugs can be directly delivered into the target cancer cells *via* using fusogenic liposomes without the risk of degradation by the endocytic pathway (Malam et al., 2009; Kube et al., 2017). The drawbacks associated with use of liposomes include spontaneous fusion of the liposome membranes, which can cause decreased drug payload concentration and increase off-target toxicity (Monteiro et al., 2018; Akbarian et al., 2020). In order to solve these problems, nanoassemblies have been designed as an effective drug delivery vehicle. The lipid-coated targeted nanoassembly composed of Col@MSN@LL-(LL-37) has proved to be a successful delivery platform (Rathnayake et al., 2020).

These findings suggested that the formulation of LL-37 with nanoparticles could be successfully used as a potential therapeutic strategy to enhance the delivery of LL-37 against cancers.

### Modification and alteration of the peptide

Another potential problem associated with LL-37 peptide is that it can be easily degraded by proteolytic enzymes present in the digestive system and blood plasma (Vlieghe et al., 2010). Susceptibility to degradation is primarily dependent on the peptide sequence. However, modification of the peptide and alteration of the sequence, such as the use of d-amino acid, sequence truncation, and modifications of C- and N-terminal, can render it unrecognizable by the various proteolytic enzymes and even influence the selectivity of the cancer cells as a basis for developing alternative cancer treatment approaches (Wang et al., 2019; Tornesello et al., 2020; Trinidad-Calderón et al., 2021). For instance, part of the LL-37 C-terminal domain, peptide sequence: FRKSKEKIGKEFKRIVQRIKDFLRNLV was found to display antiproliferative effects on human squamous cell carcinoma (Okumura et al., 2004). Moreover, a part of LL-37, KR12C: N-KRIVKLIKKWLR-C, could promote apoptosis in human breast cancer cells (Sengupta et al., 2018). The LL-37 fragments and analog peptides, such as FF/CAP18: FRKSKEKIGKFFKRIVQRIFDFLRNLV, with replacements of a glutamic acid residue and a phenylalanine at position 20, exhibited the functions of both inhibiting proliferation and promoting apoptosis in colon cancer (Kuroda et al., 2012; Kuroda et al., 2015b; Kuroda et al., 2017; Hayashi et al., 2018). Interestingly, the residues 17–32 of LL-37, abbreviated as FK-16 (FKRIVQRIKDFLRNLV) were found to induce apoptotic cell death and autophagy in the cancer cells, and these effects were even superior to that of LL-37 (Li et al., 2006; Ren et al., 2013; Zhang et al., 2019). It was observed that these peptides containing amino acid substitutions induce apoptosis in some specific types of cancer cells that have more negatively charged cell membranes than those in the normal cells, largely as compared to the original peptide. Furthermore, the variant of LL-37, obtained by cutting out both the C-terminus coil part and the N-terminus heparan sulfate binding region and replacing some positively charged amino acids with histidines, was found to display higher affinity and generic tumor selectivity than the original peptide (Capozzi et al., 2018). Specific positional Q and K mutants of LL-37 were observed to have lower hemolytic toxicities and preserved the cell-penetrating ability of human breast cancer cells (Kim et al., 2016).

### Combinatorial applications of LL-37

Interestingly, some evidence suggests that combined treatment using LL-37 and chemotherapy drugs can yield better results. For example, combinatorial application of LL-37 and etoposide exhibited significantly better antitumor effects on C6 glioma cells (Chernov et al., 2022). Compared with CpG ODN or LL-37 alone, the combination of LL-37 and CpG-ODN in the treatment of ovarian cancer can produce better antitumor effects and improve survival rates (Chuang et al., 2009). The mechanism can be expressed as the combinational use of LL-37, and CpG-ODN enhances the ability of human B lymphocytes and plasma-like dendritic cells to recognize and bind to CpG oligonucleotide and then leading to the activation of TLR-9 (Hurtado and Peh, 2010). Furthermore, the anticancer efficacy of the LL-37 fragment peptide analog was enhanced *via* linking PLGA conjugate (Mori et al., 2021). Compared with the peptide alone, the conjugate micelles were shown to effectively inhibit tumor cells and increase cell permeability in colon cancer, gastric cancer, hematologic malignancy, and oral squamous cell carcinoma. In addition, when LL37 was genetically fused with M-CSFRJ6-1 in the murine model, the antitumor immune response of the M-CSFRJ6-1 DNA vaccine was also enhanced (An et al., 2005). It suggests a possible use of LL-37 as an immune adjuvant in the gene therapy of some types of diseases, such as leukemia, Hodgkin’s disease, and many solid tumors. This practical approach not only enhances the effect of traditional anticancer drugs but also markedly reduces the dosage of peptide and potential cytotoxicity.

## Conclusion and future perspectives

Human cathelicidin LL-37 is an interesting peptide, which can display multiple functional roles and has been implicated in numerous diseases. The extensive functions of the peptide provide a scientific basis for analyzing its potential applications. The high interest in the therapeutic potential of this peptide originates from its potency against targeting bacteria. However, there is an increasing amount of evidence about the anticancer effects of LL-37. To date, the poor bioavailability, high production cost, and potential cytotoxicity have effectively limited the therapeutic use of LL-37.

Although a large number of studies have shown that the anticancer effects of LL-37 have potential applications in novel cancer treatment strategies, there remain some major challenges that need to be overcome. Particularly, as described in this review, the sensitivity of LL-37 varies among different cancer types. For instance, in colon cancer, glioblastoma, hematologic malignancy, gastric cancer, and oral squamous cell carcinoma, LL-37 can suppress proliferation and induce autophagy as well as apoptotic cell death *via* both non-membrane-based and membrane-based mechanisms. However, in other types of cancer, such as lung cancer, breast cancer, ovarian cancer, melanoma, prostate cancer, liver cancer, and skin squamous cell carcinoma, it can promote proliferation, migration, and tumorigenesis. To date, there is still no conclusive proof to explain the opposite effects of LL-37 on various cancer types. Furthermore, its selectivity and toxicity are complex. It will be very important to consider the different strategies to enhance both delivery and selectivity of LL-37 for cancer cells.

As a milestone, a phase 1 clinical trial (NCT02225366) with intra-tumoral injections of LL-37 for melanoma patients with cutaneous metastases has been completed and shown significant potency against cancer. We anticipate that research interest in the therapeutic potential of LL-37 will continue to expand, and there will be new discoveries in the near future. These achievements will reignite the hope to develop this important peptide into a novel anticancer drug suitable for clinical use.
